# An Intimate Relationship between Thyroid Hormone and Skin: Regulation of Gene Expression

**DOI:** 10.3389/fendo.2013.00104

**Published:** 2013-08-22

**Authors:** Dario Antonini, Annarita Sibilio, Monica Dentice, Caterina Missero

**Affiliations:** ^1^CEINGE Biotecnologie Avanzate, Napoli, Italy; ^2^Department of Clinical Medicine Surgery, University of Naples Federico II, Napoli, Italy; ^3^Fondazione IRCCS SDN, Napoli, Italy

**Keywords:** thyroid hormones, skin, deiodinase, regulation of gene expression, epidermal development

## Abstract

Skin is the largest organ of the human body and plays a key role in protecting the individual from external insults. The barrier function of the skin is performed primarily by the epidermis, a self-renewing stratified squamous epithelium composed of cells that undergo a well-characterized and finely tuned process of terminal differentiation. By binding to their receptors thyroid hormones (TH) regulate epidermal cell proliferation, differentiation, and homeostasis. Thyroid dysfunction has multiple classical manifestations at skin level. Several TH-responsive genes, as well as genes critical for TH metabolism and action, are expressed at epidermal level. The role of TH in skin is still controversial, although it is generally recognized that TH signaling is central for skin physiology and homeostasis. Here we review the data on the epidermis and its function in relation to TH metabolism and regulation of gene expression. An understanding of the cellular and molecular basis of TH action in epidermal cells may lead to the identification of putative therapeutical targets for treatment of skin disorders.

## Introduction

**Figure 1:**
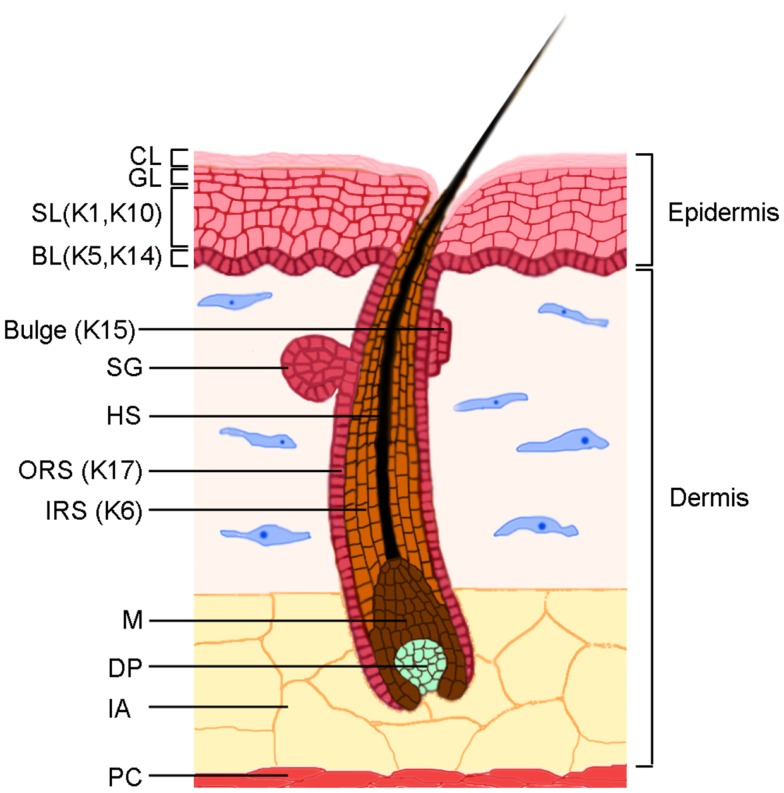
**The skin structure**. The skin is characterized by two main components, the epidermis and the dermis, which are separated by a basement membrane. The epidermis is constituted by an undifferentiated basal layer (BL) of cells that progressively differentiate in the spinous layer (SL), granular layer (GL), and the cornified layer (CL). The HF consists of an outer root sheath (ORS), an inner root sheath (IRS), and a hair shaft (HS). The epidermal stem cell compartment resides in the BL of the epidermis and in a specific region of the ORS named *bulge*. The lower part of the follicle, the hair bulb, is characterized by proliferating matrix cells (M) and by the dermal papilla (DP), which is the dermal component of the HF. The sebaceous glands (SG) are integral part of the pilosebaceous unit. The major keratins expressed in different compartments are indicated. The dermis is composed by dense connective tissues in which the fibroblasts are the main components. Intradermal adipocytes (IA) are abundant in the lower part of the dermis, above the panniculus carnosus (PC), a layer of striated muscle cells.

Skin plays a key role in protecting the body from dehydration, mechanical trauma, and microbial insults (Figure 1). The epidermis is composed of keratinocytes, and to a lesser extent of resident dendritic cells, T lymphocytes, melanocytes, and the neuroepithelial sensory Merkel cells. Dendritic cells and T lymphocytes protect skin from microbial insults, which makes skin an active immune organ [reviewed in (1)]. The epidermis receives nutrients from blood vessels in the underlying dermis, which contains several types of cells, including fibroblasts that produce proteoglycans, collagen, and elastic fibers, as well as resident dendritic cells, macrophages, mast cells, and lymphocytes. Hair follicles (HF) and sebaceous glands, two epidermal appendages, are embedded in the dermis and are separated from the dermis by the basement membrane, whose components are secreted by epidermal keratinocytes and dermal fibroblasts.

Being the largest organ of the human body, skin is also an important peripheral neuro-endocrine-immune organ that is closely related to endocrine systems, thus contributing to the maintenance of peripheral hormonal homeostasis. Specialized cells in the skin produce and respond to multiple neurotransmitters, neuropeptides, and hormones thereby making skin a central player in endocrine homeostasis.

This review focuses on the gene expression programs activated in the skin, and in particular in the interfollicular epidermis, during embryogenesis and in adult life in relationship with a specific class of hormones secreted by the thyroid gland, namely the triiodothyronine (T3) and thyroxine (T4).

## Transcriptional Control of Epidermal Development and Establishment of Barrier Function

The barrier function of the epidermis is established during embryogenesis and is the result of a complex and coordinated stratification program. During mouse embryogenesis, cells of the surface neuroectoderm are committed to an epidermal fate at embryonic day 8.5 (E8.5), and progressively acquire expression of basal cell markers. Their commitment is regulated in large part by cross-regulation between WNT and BMP signaling. WNT signaling suppresses epidermal cell fate in favor of the neural fate in the dorsal region of the developing embryo, whereas BMP signaling is predominant in the ventral region of the embryo and it is thought to act as epidermal inducer (2). Given the crucial role of BMP signaling in skin development, it is interesting to note that both epidermal cells and dermal fibroblasts express several BMP ligands and BMP receptors. Treatment of these cell types with BMP induces cell-type-specific changes in gene expression programs, which are likely to contribute to the complex effects of BMPs in the developing skin and in skin homeostasis (3).

The first ectodermal-specific marker expressed as early as E8.5 is the transcription factor p63 is a master regulator of stratified epithelia (4–8) that continues to be expressed during embryonic skin development and in the basal proliferative layer in postnatal life also through a positive autoregulatory loop (4). Studies with zebrafish demonstrated that p63 is a direct target of BMP signaling and blocks neural specification (9). A similar role in maintaining epidermal cell fate has been demonstrated in mammalian cells, where p63 depletion results in ectopic expression of neural and mesenchymal genes (10–12). Interestingly, p63 positively regulates BMP signaling through direct activation of BMP7 and repression of SMAD7 (11–13).

During epidermal morphogenesis, p63 expression is followed by regional expression of keratins K5 and K14 at E9.5 (14). Their expression is regulated directly by p63 itself (15, 16), and by other crucial regulators expressed in the early surface ectoderm including transcription factors of the AP-2 family (14, 17, 18).

Among its multiple functions, p63 plays a crucial role in sustaining epidermal progenitor expansion by directly induction of the fibroblast growth factor receptors Fgrf2 and Fgfr3 (19), and by suppression of the cell cycle inhibitors p21 (Cdkn1a), INK4a (Cdkn2a), ARF (Cdkn2d), and the microRNA miR-34a (20–25). During development, a rapid increase in embryo size is accompanied by frequent symmetric divisions of epidermal progenitors thereby increasing surface area. Stratification occurs through asymmetric cell division starting from E13.5, when the spinous layer of initially proliferating cells expressing keratins K1 and K10 is formed above the basal layer. Loss of this proliferative capacity is associated with maturation into spinous cells, which subsequently undergo further maturation into granular and cornified cells, and by E17.5 the epidermal barrier is fully formed and the skin becomes impermeable. Notch signaling is required for induction of the spinous layer markers, keratins K1 and K10, and involucrin (26–28). Cross-talk between the p63 and Notch signaling pathways has been associated to the commitment of keratinocytes to undergo terminal differentiation, because p63 represses a subset of Notch functions, whereas Notch signaling suppresses p63 expression (22). Interestingly, p63 depletion inhibits expression of Notch1 itself and its ligand Jag1 (13, 29). Therefore, during normal development, Notch is necessary and sufficient to induce many aspects of differentiation, and the cross-talk between p63 and Notch signaling is essential to control stratification during development.

Also Irf6, Ikkα, and Klf4 are crucial regulators of epidermal development and keratinocyte differentiation. Ikkα, a p63 target gene (30, 31), is required to exit from the basal cell compartment, although its function in the switch between basal and suprabasal cell fate is not cell-autonomous (32–35). Irf6-deficient mice have a hyperproliferative epidermis that results in the expansion of the basal and spinous layer, loss of granular layer, and defective epidermal barrier (36, 37). Interestingly, Irf6 is induced during differentiation via p63- and Notch-dependent mechanisms (38–40), whereas Irf6 negatively regulates p63 levels by inducing proteasome-mediated degradation (40). Granular layer and epidermal barrier formation is also controlled by the transcription factor Klf4 as revealed by studies in mice lacking or overexpressing Klf4 (41, 42). Thus, these transcription factors orchestrate the differentiation program during development although their interconnections have not been fully established.

## Structure of Mature Epidermis

In adult epidermis, keratinocytes undergo a continuous program of terminal differentiation, starting from the basal layer and moving outward to form the suprabasal layers. Epidermal cells are protected from physical trauma by specialized intermediate filaments (IF) and specific cell-basement membrane and cell–cell junctions. In epidermal keratinocytes, IF are mainly constituted by keratins and are particularly abundant; in fact, they account for up to 85% of the total protein content of the cells. Keratin mutations or depletions cause cells to become fragile and prone to ruptures [reviewed in (43)]. Keratins K5 and K14 are expressed in the basal layer of the interfollicular epidermis and of the HF, as well as in the basal layer of other stratified epithelia. K15 is a less abundant component of the basal IF, and is thought to compensate at least in part for loss of K14 by assembling with K5 (44). Although the function of K15 in the skin has not been fully elucidated, it is an intriguing molecule since it is more abundant in the newborn interfollicular epidermis and in the adult HF bulge (45) where in cells with higher proliferative potential and stem cell like properties. Under physiological conditions, K5/K14 are substituted by K1/K10 in the spinous layer, whereas under hyperproliferative conditions, including inflammation, acute injury, psoriasis, and carcinoma, the HF keratins K6, K16, and K17 are induced suprabasally in the epidermis [(46) and reference therein]. Beside its role in the IF, K17 regulates cell growth and size by inducing protein synthesis through the Akt/mTOR-dependent pathway (47). In a more indirect fashion, K17 expression in the epidermis is also involved in inflammation-dependent cell proliferation by inducing an immune response (48).

Basal keratinocytes are anchored to the basement membrane by integrins and by specialized junctions, hemidesmosomes constituted by integrins, and other proteins that connect them to the actin cytoskeleton [reviewed in (49)]. Epidermal cells are tightly packed one to the other by cell–cell junctions, namely desmosomes, adherens junctions, and tight junctions [reviewed in (50)]. Adherens junctions are intercellular structures that couple intercellular adhesion to the actin cytoskeleton and are formed by two cell adhesion receptor complexes, the classical cadherin/catenin complex and the nectin/afadin complex, both of which can link to the actin cytoskeleton.

Desmosomes form a robust network among adjacent cells conferring strength and resiliency to the epidermis, with basal and suprabasal cells expressing different sets of specific desmosomal proteins [reviewed in (51)]. Desmosomal cadherins are transmembrane proteins that form stable associations with desmosomal cadherins in adjacent cells and are linked inside the cell to the keratin cytoskeleton. Interestingly, several components of hemidesmosomes and desmosomes are under the tight transcriptional control of p63 (29, 52–55). By controlling several adhesion molecules, p63 is essential for cell adhesion in stratified epithelia and its absence leads to epidermal fragility (29, 53).

The final step in epidermal differentiation involves the formation of the epidermal barrier, which is characterized by the formation of cornified cell envelopes constituted by proteins cross-linked by specific enzymes into a rigid scaffold and by lipids covalently attached to the exterior surface. The barrier function of the epidermis is also maintained by tight junctions, and the first functional evidence that epidermal barrier function requires a tight junction component came from claudin-1-deficient mice, which die of massive transepidermal water loss due to impaired barrier function of the granular layer (56).

## Thyroid Hormone Action and Metabolism

The thyroid hormone (TH) is a key element in the endocrine control of epidermal development and function [reviewed in (57)]. Clinical evidence (58–63) as well as studies conducted in hypothyroid mice and rats (64–66) suggest that TH is involved in epidermal proliferation and differentiation, hair growth, and wound healing besides affecting the function of dermal fibroblasts. The importance of TH in skin was first shown in lower vertebrates. In Amphibian metamorphosis, the skin is transformed from a bilayered non-keratinized epithelium into a stratified, keratinized epidermis (67).

To exert its functions, TH must overcome several checkpoints, namely TH transporters, TH-metabolizing enzymes (deiodinases), TH receptors (TRs), and their interactions with co-repressors and co-activators [reviewed in (68)]. In the bloodstream, the steady-state level of TH concentration is regulated by the hypothalamic-pituitary-thyroid (HPT) axis. Hypothalamic thyroid releasing-hormone (TRH) stimulates thyrotrophic cells in the anterior pituitary to produce thyroid stimulating-hormone (TSH). In turn, TSH induces the production of pro-hormone thyroxine (T4) and – to a lesser extent – the active form triiodothyronine (T3) by the thyroid gland. Unexpectedly the expression of fully functional proteins typical of the HPT axis, and in particularly of the TSH receptor, the TRH and the THR receptor has been found in human skin and in the HF (58, 69–72). Both, TRH and TSH exert several regulatory effects on skin-specific gene expression and various elements of the HPT axis are transcribed by human skin cell populations (69, 73) suggesting that normal human skin might represent a source and extrathyroidal target of TRH and TSH. Epidermal expression of TSH is up-regulated by TRH and repressed by THs, demonstrating that intraepidermal TSH expression is regulated by the classical endocrine controls that determine the systemic HPT axis. Moreover, TSH treatment of organ-cultured human skin induces expression epidermal differentiation markers, namely involucrin, loricrin, and keratins 5 and 14 (69). Among other functions, TSH or TRH treatments of scalp human skin *ex vivo* up-regulate mitochondria biogenesis (74, 75). In addition, TSH stimulation up-regulates the transcription of classical TSH target genes thyroglobulin and thyroid transcription factor-1 (Nkx2.1) and enhances cAMP production into the culture medium (58), documenting that the TSH receptor expressed by normal human scalp HF *in situ* is functionally active. Behind the canonical TSH-dependent regulation, recently it has been demonstrated that the TSHR can also be activated by a newly discovered glycoprotein hormone, known as thyrostimulin (76). This hormone is composed of a dimer of unique α 2 and β 5 subunits. Interestingly, both subunits have been documented to be expressed in different tissues including the skin, suggesting a functional role for TSHR signaling via locally produced thyrostimulin in the skin (77). Collectively, these data identify non-classical functions of TRH and TSH-mediated signaling in skin, suggesting that these hormones represent novel players in skin physiology and in human epithelial cell biology and encourage new studies to reveal molecular mechanisms underlying TH action in skin and its appendages.

Once TH enters the bloodstream, a low amount of TH, not bound to circulating transport proteins, is free to act on target cells. The initial step in the activation of TH is its transport across the cell membrane that is mediated by different types of TH-transporting proteins (78–80). These transporters are differentially expressed in tissues in a developmental and cell-type-specific fashion and, while most of them accept a variety of ligands, others have elevated substrate specificity (81, 82). The latter include monocarboxylate transporters 8 and 10 (MCT8 and MCT10) (83, 84), organic anion transporters 2 and 3 (Oatp2 and Oatp3), and l-type amino acid transporters (Lat1 and Lat2). At present, little is known about TH-transporter expression in skin and/or epidermis. The uptake of T3 and T4 is much lower in skin fibroblasts from patients with a MCT8 mutation than in controls, which indicates that MCT8 is expressed in these cells (85).

Within target cells, TH is metabolized by the action of deiodinases, three thioredoxin fold-containing selenoenzymes. These enzymes metabolize TH in a stage- and tissue-specific manner by a mono-deiodination reaction that involves two distinct pathways. Type I and II deiodinases (D1 and D2) convert the inactive pro-hormone T4 to the active form T3 – a process that increases circulating T3 levels and the availability of the active hormone for nuclear receptors [reviewed in (86)]. D1 regulates circulating T3 levels, whereas D2 acts essentially at the intracellular level (87).

In contrast, type III deiodinase (D3) inactivates TH by converting T4 and T3 to the inactive metabolites reverse T3 (rT3) and T2, respectively. All three deiodinases are integral membrane proteins that share a conserved region ∼15 amino acids long within the active center that encodes a selenocysteine that enables the deiodinases to exert enzymatic activity. The subcellular localization differs among the three enzymes, and this affects their systemic versus cellular contributions to TH homeostasis (88). Notably, the combined actions of D2 and D3 are viewed as a cell-autonomous pre-receptoral mechanism that controls TH signaling in a time- and tissue-specific manner without affecting serum hormone concentrations (89, 90). Often the activities of the D2 and D3 enzymes are finely tuned and oppositely regulated in different cell contexts to ensure the correct balance between the activating/inactivating deiodinases (91, 92); Histone H3 demethylating enzyme (LSD-1) and Foxo3 are critical regulators of this balance in muscle (93), while their role in skin has not been established.

**Figure 2:**
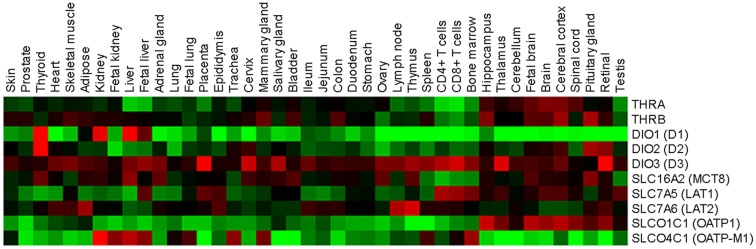
**Relative expression profiles of TH regulators and effectors in normal human tissues**. Forty-two normal human tissues were analyzed for TH regulators and effectors using a custom high-density microarrays (GEO accession: GSE14938) (126). Results are expressed as heat map of fold changes (log10 ratio) relative to the pool of tissues.

Rat skin was the first organ shown to be an active site for the inner ring mono-deiodination of thyroxine to T3 (94). Subsequently, it was discovered that newborn and adult human epidermal keratinocytes in culture are able to convert T4 to T3 by D2 (94, 95), which suggests that a finely regulated TH metabolism is present at skin level. In addition, various studies showed that D3 protein is present in both mouse and human skin. D3 is abundant in murine epidermis and its expression is finely regulated during murine cutaneous development (96, 97). D3 expression first appears in the mouse epidermis at E13.5 just before stratification, and it is highly expressed in the epidermal layers and in the HF at E17.5 and at P0. Moreover, D3 expression is elevated in the growing phase of the HF cycle in the most external portion of the follicle, while it is less detectable in the regressing and resting phases of the HF cycle (96). This is in agreement with the sustained expression of D3 observed in various cancers, including basal cell carcinoma (BCC) and colon cancers, in which D3 is required to facilitate the proliferation of neoplastic cells (9, 90, 96). D2 and D3 transcripts are expressed also in whole human skin biopsies, and in epidermal and dermal cells, although it remains to be established how these genes are transcriptionally regulated in these cells (63, 72). These findings strongly suggest that the D2 and D3 deiodinases are crucial components in the control of intracellular TH in skin, whereas D1 is not expressed in skin (Figure 2).

Once the active hormone T3 is present inside the cells, it can enter the nucleus and reach the TRs, a family of ligand-dependent transcription factors that enhance or inhibit the expression of target genes by binding to specific DNA sequences, known as TH response elements (TREs). Thyroid hormone receptors exist in two isoforms, TRα and TRβ, which are encoded by the THRA and THRB genes, respectively [reviewed in (68, 98)]. Also TR expression is specifically modulated and both isoforms express several splicing products that are differently expressed in development and in adult tissues (99) (Figure 2). The TR-TH complex occurs often as a homodimer but also as a heterodimer with the retinoid-X receptor (RXR) (100–102). The ligand availability and concentration of the TR complex in the nucleus ultimately define the level of TR transcription activity. In the classical model of transcriptional regulation by TH, nuclear receptor co-repressors (e.g., NCoR-SMRT) are bound to the TR complex in the unliganded state (68, 98). The binding of T3 to TR induces structural changes, and the co-repressors are released and replaced by co-activators (e.g., SRC/p160 or TRAP/DRIP complex) that modify chromatin structures. This complex ultimately recruits RNA polymerase II and leads to transcriptional activation of responsive genes (68). According to a debated non-classical mechanism of TH action, T3 and T4 exerts also a non-genomic effect. Specifically, TH signaling results from the binding of T3 and T4 to a membrane integrin, αvβ3, which leads to activation of the PI3K and MAPK transduction pathways and in turn increases target gene transcription [reviewed in (103)].

TH receptors are expressed in skin (104–106) and play a functional role as demonstrated by the phenotype of the double TRα and TRβ deficient mice (107). Epidermal cell proliferation and cyclin D1 expression is reduced in the interfollicular epidermis of mice lacking both TRα and TRβ under basal conditions and upon treatment with the phorbol ester TPA or with retinoic acid (107, 108). In parallel with reduced epidermal cell proliferation, expression of proinflammatory cytokines coupled with signs of skin inflammation is induced in double TRα and TRβ deficient skin. Further investigation, possibly with conditional mouse models, are required to determine whether reduced cell proliferation in the epidermis is a cell-autonomous phenotype.

## Regulation of Gene Expression by Thyroid Hormone in the Skin Under Physiological and Pathological Conditions

In humans, thyroid dysfunction is associated with alterations in skin architecture and homeostasis [reviewed in (62)]. In hyperthyroid individuals, skin often presents with some of the following symptoms: softness, perspiration, heat, itching, generalized pruritus, chronic urticaria, vitiligo, and diffuse skin pigmentation (109, 110). In addition, the epidermis is usually thinner than normal. In hypothyroid subjects, the skin is dry, cold, and rough. The epidermis is hyperkeratotic, alopecia may develop, and there is diffuse myxedema (110).

The molecular mechanisms at the basis of these cutaneous symptoms have not been clarified, but there is evidence implicating TH in the molecular control of epidermal differentiation and barrier formation during development (111, 112), hair growth, sebum production, keratinocyte proliferation, and keratin gene expression (62, 111–113). Indeed, TH action is crucial for the balance between proliferation and differentiation in normal and pathological conditions, including epidermal regeneration (65) and cutaneous cancer (96).

Many keratins have been identified as TH-responsive genes [reviewed in (61)]. In amphibian metamorphosis, TH is required for skin changes and correlates with the expression of adult keratins and the loss of embryonic keratins (114). In mammalian epidermis and in the HF, TH regulates a number of keratins, including K5, K14, K6, K16, and K17 (63, 64, 113, 115, 116). Similarly, TRα and TRβ can regulate either positively or negatively the expression of selected keratins in cultured cells (61, 117–119). Another TH-responsive keratin is K15; in fact, its promoter activity is significantly induced by the presence of T3 (119), and physiological concentrations of TH induce its expression in epithelial stem cells of adult human scalp HFs (120).

Various studies suggest that TH is also a key regulator of several ubiquitously expressed genes involved in keratinocyte proliferation and differentiation, although TH function is skin is still controversial. The TH analog (TRIAC) stimulates epidermal thickening in mice and promotes human keratinocyte proliferation by activating cyclin D1 expression and promoting entry into the S phase of the cell cycle (121). Hair matrix keratinocytes treated with TRH and T4 show increased proliferation and inhibited apoptosis (63, 71). In contrast, Tiede et al. demonstrated that THs reduce proliferation, cyclin D1 expression, and induce apoptosis of isolated K15-positive HF stem cells (120).

An anti-proliferative function of THs in the skin has been shown also in mouse keratinocytes and in BCC, the most frequent human cancer, which originates from epidermal stem cell compartment in response to aberrant constitutive activation of the Sonic Hedgehog (Shh) pathway (122). Shh, through the transcriptional factor Gli2, directly induces local inactivation of TH by up-regulation of D3 (the TH action terminator) in proliferating keratinocytes and in mouse and human BCCs, thus resulting in increased cyclin D1 and keratinocyte proliferation. Consistently, D3 knockdown in BCC cells causes a drastic reduction of cellular proliferation and a reduction in the growth of BCC xenografts in nude mice *in vivo* (96). On the other hand, Hedgehog signaling promotes reduction of the TH signaling by D2 degradation (the principal TH activator) via the E3 ubiquitin ligase adaptor (WSB-1) in embryonic structures during chicken development (96).

Several gene expression profiling studies have identified TH-responsive genes in human skin fibroblasts and in dermal cells (Table 1). For instance, members of the aldo-keto reductase (AKR) family, a member of the RAS oncogene family (RAB3B), PFKP, collagen (COLVIA3-COLVIIIA1), solute carrier family 16 member 3 (SLC16A3), enolase 1 (ENO1), the hypoxia-inducible factor (HIF)-1α, a calcineurin inhibitor ZAKI 4α (also known as Down syndrome critical region 1 L1), and glucose transporter 1 (GLUT1) are all increased in human skin fibroblasts treated with T3 (123). In the same condition, TH mediates the down-regulation of fibroblast growth factor 7 (FGF7), a potent stimulator of epidermal proliferation, and alcohol dehydrogenase 1B (ADH1B) (123). In dermal cells, T3 inhibits the synthesis of hyaluronate (HA), which is a type of glycosaminoglycan, by down-regulating HA synthase 2 (HAS2) (124). All these TH-responsive genes exert a variety of regulatory functions in development and metabolism. Lastly, a set of genes associated with cell-basement membrane cell adhesion (integrin beta4, plectin, and collagen XVII) is suppressed in T3-treated human epidermal keratinocytes (125).

**Table 1:** **Regulation of gene expression by TH in skin cells**.

Keratin genes	TH regulation	Cellular system	Reference
K5	↓	Normal human epidermal keratinocytes	(115)
K14	↓	Normal human epidermal keratinocytes, human HFs	(63, 115)
K6	↑	Human epidermal keratinocytes (HaCat), human HFs	(63, 64)
K16	↑	Human epidermal keratinocytes (HaCat)	(64)
K17	↓, ↑	Human epidermal keratinocytes (HaCat)	(64, 115)
K15	↑	Human epithelial HF stem cells	(119, 120)
Other (TH-responsive) genes
Cyclin D1	↑, ↓	Mouse/human epidermal keratinocytes, human epithelial HF stem cells	(96, 107, 120, 121)
P19, p27	↑	Mouse epidermal keratinocytes	(107)
AP1, NF-KB, STAT3	↓	Mouse epidermal keratinocytes	(107)
TGF-β2	↓	Mouse epidermal keratinocytes	(107)
AKR	↑	Human skin fibroblasts	(123)
RAB3B	↑	Human skin fibroblasts	(123)
COLVIA3-COLVIIIA1	↑	Human skin fibroblasts	(123)
ENO1	↑	Human skin fibroblasts	(123)
HIF-1α	↑	Human skin fibroblasts	(123)
ENO1	↑	Human skin fibroblasts	(123)
ZAKI 4α	↑	Human skin fibroblasts	(123)
GLUT-1	↑	Human skin fibroblasts	(123)
FGF7	↓	Human skin fibroblasts	(123)
ADH1B	↓	Human skin fibroblasts	(123)
HAS2	↓	Dermal cells	(124)
Integrin β4	↓	Human epidermal keratinocytes	(125)
Plectin	↓	Human epidermal keratinocytes	(125)
COLXVII	↓	Human epidermal keratinocytes	(125)

In conclusion, we provide an overview of recent data about the intricate mechanisms controlling intracellular TH action in skin and in particular in the epidermal compartment. We focus particularly on the role of TH metabolism and deiodinases in pathophysiological settings. Despite progress in our understanding of the function of these enzymes, much more remains to be discovered. In particular, the tissue-specific deletion of individual, and combined, deiodinases at epidermal level will certainly shed light on their role in the epidermal compartment in normal and pathological conditions. The concept that a finely tuned TH concentration is essential in the control of proliferation versus differentiation raises the possibility of interfering with such mechanisms for therapeutic purposes. Unraveling these complex interactive mechanisms is an exciting challenge for the future and a promising source of information to determine how to regulate TH action in skin.

## Conflict of Interest Statement

The authors declare that the research was conducted in the absence of any commercial or financial relationships that could be construed as a potential conflict of interest.
